# Development of polymorphic microsatellite markers by using de novo transcriptome assembly of *Calanthe masuca* and *C. sinica* (Orchidaceae)

**DOI:** 10.1186/s12864-018-5161-4

**Published:** 2018-11-06

**Authors:** Chao Hu, Hongxing Yang, Kai Jiang, Ling Wang, Boyun Yang, Tungyu Hsieh, Siren Lan, Weichang Huang

**Affiliations:** 1 Shanghai Key Laboratory of Plant Functional Genomics and Resources, Shanghai Chenshan Botanical Garden, Shanghai, 201602 China; 2 Shanghai Chenshan Plant Science Research Center, Chinese Academy of Sciences, Shanghai Chenshan Botanical Garden, Shanghai, 201602 China; 3 Institute of Botany, Chinese Academy of Sciences, Beijing, 100093 China; 4 School of Life Science, Nanchang University, Nanchang, 330031 China; 5 Shanghai Institutes for Biological Sciences, Chinese Academy of Sciences, Shanghai, 200031 China; 6 College of Landscape, Fujian Agriculture and Forestry University, Fuzhou, 350002 China

**Keywords:** Polymorphic microsatellite, Next-generation sequencing, Population genetics, Divergence time

## Abstract

**Background:**

*Calanthe masuca* and *C. sinica* are two genetically closely related species in Orchidaceae. *C. masuca* is widely distributed in Asia, whereas *C. sinica* is restricted to Yunnan and Guangxi Provinces in southwest China. Both play important roles in horticulture and are under the pressure of population decline. Understanding their genetic background can greatly help us develop effective conservation strategies for these species. Simple sequence repeats (SSRs) are useful for genetic diversity analysis, presumably providing key information for the study and preservation of the wild populations of the two species we are interested in.

**Results:**

In this study, we performed RNA-seq analysis on the leaves of *C. masuca* and *C. sinica*, obtaining 40,916 and 71,618 unigenes for each species, respectively. In total, 2,019/3,865 primer pairs were successfully designed from 3,764/7,189 putative SSRs, among which 197 polymorphic SSRs were screened out according to orthologous gene pairs. After mononucleotide exclusion, a subset of 129 SSR primers were analysed, and 13 of them were found to have high polymorphism levels. Further analysis demonstrated that they were feasible and effective against *C. masuca* and *C. sinica* as well as transferable to another species in *Calanthe*. Molecular evolutionary analysis revealed functional pathways commonly enriched in unigenes with similar evolutionary rates in the two species, as well as pathways specific to each species, implicating species-specific adaptation. The divergence time between the two closely related species was tentatively determined to be 3.42 ± 1.86 Mya.

**Conclusions:**

We completed and analysed the transcriptomes of *C. masuca* and *C. sinica*, assembling large numbers of unigenes and generating effective polymorphic SSR markers. This is the first report of the development of expressed sequence tag (EST)-SSR markers for *Calanthe*. In addition, our study could enable further genetic diversity analysis and functional and comparative genomic studies on *Calanthe*.

**Electronic supplementary material:**

The online version of this article (10.1186/s12864-018-5161-4) contains supplementary material, which is available to authorized users.

## Background

**Fig. 1:**
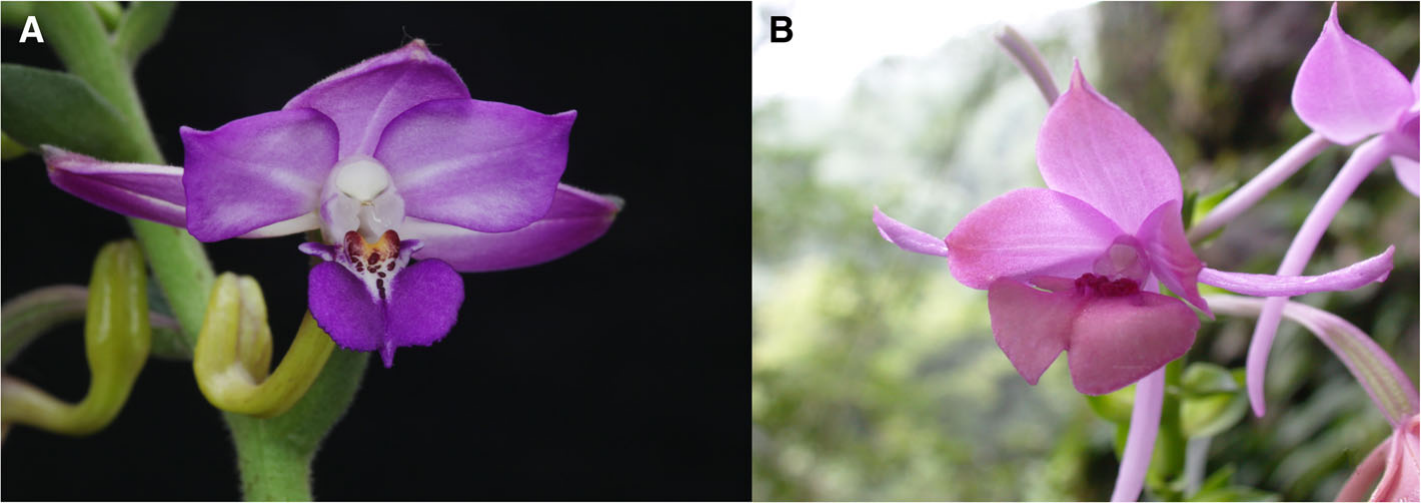
Flowers of *C. masuca* and *C. sinica*. **a** The flowers of *C. sinica.* **b** The flowers of *C. masuca*

*Calanthe* R. Br. is a large genus in Orchidaceae, comprising 207 [1] epiphytic and terrestrial species. These species are widely distributed across the pantropical area, and southeast Asia is one of the diversity centres of *Calanthe* [1]. Subgenus *Calanthe* section *Calanthe* series *Sylvatica* of genus *Calanthe* includes 14 species widely distributed from southeast Asia to Africa, with only one species, *Calanthe sylvatica* (Thouars) Lindl., distributed on the African continent. Few studies have focused on *Sylvatica* species, although these species are of high horticultural value and play an important role in breeding. Two of these species are from China – the widely distributed *Calanthe masuca* (D. Don) Lindl. and the narrowly distributed *C. sinica* Z. H. Tsi (Fig. 1), which is restricted to southwest China. A molecular phylogeny based on both nuclear and cpDNA markers showed that these two species are genetically closely related [2]. These two species have the same number of chromosomes (2n = 40) [3, 4] and perhaps large genomes (the genus average C-value is ~ 11.23 pg) [5]. *C. sinica* is assessed as CR (critically endangered) according to IUCN Red List Categories and Criteria. Recent years have seen rapid population decline for these species, likely due to destruction of habitat and collection. Therefore, research on these species is necessary for further protection strategies. With these closely related species, it is possible to investigate the relationship between *Calanthe* species from Asia and Africa as well as the driving force underlying the divergence between *Sylvatica* species.

Molecular markers are important genetic tools for population genetic studies. With advantages of hypervariability, multi-allelic nature, co-dominant inheritance, reproducibility, wide genomic distribution, chromosome-specific location, amenability to automation and high-throughput genotyping [6], simple sequence repeats (SSRs) play an important role in plant genetics and breeding. Currently, next-generation sequencing (NGS) technology provides a convenient and easy way to obtain large-scale EST-SSRs from RNA-sequencing (RNA-seq). SSRs can be predicted after the gain of transcripts from NGS methods [7–9]. This technique has been widely used in plants such as *Myrica rubra, Ocimum, Vigna angularis* [10–12] and Orchidaceae [13, 14]. However, the normal method of screening for polymorphism by random selection of SSRs after they have been designated is expensive and time consuming. To solve this problem, Vukosavljev et al. [15] provided a convenient way to screen reads from multiple genotypes for repeats that showed the most length variants, which were subsequently developed into markers rather than simply presented in unfiltered results. This method was effective.

In this study, transcriptome sequencing, de novo assembly and gene annotation were performed on *C. masuca* and *C. sinica*, followed by searching for SSRs based on pairwise orthologue relationships, with the purpose of (1) screening out some polymorphic SSRs for further study of *C. masuca*, *C. sylvatica*, *C. sinica* and other species (2). The results of this study will provide transcriptome information for further functional comparative genomics research and SSR-based genetic linkage mapping in *Calanthe*.

## Methods

### Materials, total RNA extraction and sequencing

Leaves of *Calanthe masuca* and *C. sinica* grown in the nursery of Shanghai Chenshan Botanical Garden were used as the materials for RNA extraction and further analysis. The cultivation and sampling times of the two species were the same to ensure reliable comparability. Further treatments were carried out by Shanghai Majorbio Bio-pharm Technology Co., Ltd. (Shanghai, China). Young and mature leaves from each species were collected in liquid nitrogen, then total RNA of was isolated using a Trizol reagent (Invitrogen, CA, USA) according to the manufacturer’s instructions, and equal amounts of extracted RNA were pooled together. A cDNA library for each RNA-seq was obtained using a TruSeqTM RNA sample prep Kit (Illumina Inc., San Diego, CA, USA) following the manufacturer’s instructions. Subsequently, the cDNA was sequenced using a HiSeq 4000 (Illumina, San Diego, CA, USA) to obtain sequences of ~ 150 bp from both ends of each cDNA.

### Illumina sequencing, data filtering, de novo assembly and unigene annotation

Raw reads generated by the Illumina HiSeq platform (submitted to the NCBI database under accessions SRR5478842 and SRR5478843) were first processed to obtain clean reads by trimming off adaptor-ligated regions and low-quality bases (Q < 20) using SeqPrep [16] and Sickle [17]. De novo transcriptome assembly was performed based on cleaned and qualified reads using the short-read assembly program Trinity [18] with default parameters. The longest transcripts were defined as unigenes. Putative unigenes were compared against the NCBI protein non-redundant (NR) protein database and the Swiss-Prot, Pfam and KEGG databases with an E-value cut-off of 1E-5 using BLASTx (BLAST + 2.2.25 package) [19]. Gene ontology (GO) annotations describing biological processes, molecular functions and cellular components for each unigene were obtained by carrying out the program Blast2GO v.2.6.0 [20]. Kobas [21] was recruited to perform the KEGG metabolic pathway annotation.

### Orthologous gene identification and evolutionary rate calculation

Orthologous gene pairs between *Calanthe masuca* and *C. sinica* used for primer screening were identified by the method of reciprocal best hits (RBH) based on the results of the Basic Local Alignment Search Tool (BLAST) [22–25]. As the two species were phylogenetically closely related, nucleotide sequences instead of peptide sequences of the unigenes were used to run a BLAST search to improve accuracy. A python script was developed to retrieve the RBH from the results of reciprocal BLASTn (BLAST+ v 2.2.25 package), for which the cutoff was set as e-value = 1E-5.

Orthologous gene groups were defined using the program Inparanoid with default settings (version 4.1; [26]). *Phalaenopsis equestris* (Schauer) Rchb. f. was used as the outgroup species [27]. The rate of non-synonymous substitutions per non-synonymous substitution site (Ka), the rate of synonymous substitutions per synonymous substitution site (Ks), and the ratio of non-synonymous to synonymous substitution rates (Ka/Ks) for each orthologous gene pair between *C. masuca* and *C. sinica* were calculated using the method ‘GMYN’ implemented in the toolbox KaKs_Calculator (version 2.0; [28]). To calculate the Ka and Ks values, the peptide sequences of each pair of orthologous genes were first aligned using the program MAFFT [29] with default settings and were then back-translated into nucleotide sequence alignment using the program PAL2NAL [30].

### GO enrichment analysis

GO enrichment analysis was performed using the R package topGO [31], with Fisher’s exact test as the method of enrichment evaluation, all unigenes with GO annotation as the whole list of background genes, and other parameters as default.

### EST-SSR markers validation

The software Msatcommander [7] was run on the assembled transcript sequences to find SSR sites and generate primers. The unigenes of two transcriptomes were used to run Msatcommander with primer sets subsequently designed automatically. SSR loci with variations between two species were then screened out in two steps. First, sequence pairs containing SSRs were extracted from the Msatcommander results based on one-to-one correspondence by RBH search. Second, sequences containing the same SSR type (including reverse complement repeat sequences) but with different repeat numbers were selected by comparing the information of microsatellite repeats for each pair of orthologs. The primers for mononucleotide SSRs were then excluded. We only took the primer list of *C. masuca* as a result by considering that primers identified from two orthologous unigenes will yield the same product.

### Development and evaluation of EST-SSR markers

Genomic DNA was isolated from silica-gel-dried leaves using the DNAsecure Plant kit (TIANGEN Inc. Beijing, China) following the manufacturer’s protocol and stored in TE buffer (pH = 8.0). DNA purity and concentration were measured with a NanoDrop 2000c UV-Vis spectrophotometer (Thermo Fisher Scientific Inc., USA). DNA was adjusted to a final concentration of 30 ng/ml and stored at − 20 °C until use.

The primers of the selected SSR alleles were synthesized and amplified, and an M13 (− 21) sequence was added to the forward primers of these primers. An additional primer labelled with fluorescent dye (5′FAM, 5′HEX, or 5′ROX), M13 (− 21), was also synthesized. All primers were synthesized by Sangon Biological Engineering Technology & Service Co. (Shanghai, China). Amplification was carried out in a Thermocycler Mastercycler Gradient (Eppendorf, Germany) using a 20 μl reaction system containing 10 μl of 2× Taq PCR Master Mix (TIANGEN Inc. Beijing, China), 8 μl of ddH2O, 0.2 μl of forward primer (10 μM), 0.4 μl of reverse primer (10 μM) and 0.4 μl of fluorescent dye-labelled M13 primer (10 μM), and 1 μl of target DNA template (0.5 ng/μl). First, PCR was performed for every pair of primers to determine the optimal annealing temperature following Schuelke [32]: 5 min denaturation at 94 °C; 30 cycles of 30 s at 94 °C, 45 s at 53–65 °C, and 45 s at 72 °C; 8 cycles of 30 s at 94 °C, 45 s at 53 °C, and 45 s at 72 °C; a final 10 min extension at 72 °C; and 4 °C hold for storage. After determining the optimal annealing temperature of each primer, amplification was performed.

The PCR products were mixed according to the different fluorescence colour (6-FAM:HEX:ROX = 1:1:1) and then analysed on an ABI 3730 with internal size standard GS500 (GeneScanTM, Applied Biosystems, USA). Allele binning and calling were conducted using Software GeneMarker® V2.2.0 (SoftGenetics, USA). The number of alleles (*Na*), observed heterozygosity (*Ho*), gene diversity (expected heterozygosity; *He*), and polymorphism information content (PIC) for each of the EST-SSR markers were calculated using Cervus software [33].

Before screening for polymorphisms of SSR primers in multiple individuals, all selected primers were first amplified using a single sample, JX1 (*C. masuca*), and only well-amplified SSR primers were used for further testing. Then, we selected eight individuals from each population collected from Yunnan, Jiangxi, Napo, and Guangxi in China (Additional file 1) for polymorphic analysis. Finally, we chose primers with high PIC values to amplify 72 other individuals (24 from Jiangxi, 2 from Napo, 11 from Yunnan, 15 from Taiwan and 20 from Jinxiu Guangxi). In total, 96 accessions from five populations of three species were used for genetic diversity analysis, including three *Calanthe masuca* populations (JX, GXJX, TW), one *C. sinica* population (GXNP) and one *C. triplicata* population (YN) (Additional file 1).

### Universality evaluation of SSR primers

The universality of the developed SSR primers in *Calanthe* was calculated by in silico PCR. The SSR primers of *C. masuca* and *C. sinica* were searched against the unigenes of each other and against the genome sequences of *P. equestris* [27] and *Dendrobium catenatum* Lindl. [34]. e-PCR [35] version 2.3.9 was carried out with the SSR primers as queries and the unigenes as databases with no mismatch allowed.

## Results

### De novo sequence assembly and orthologous genes

**Fig. 2:**
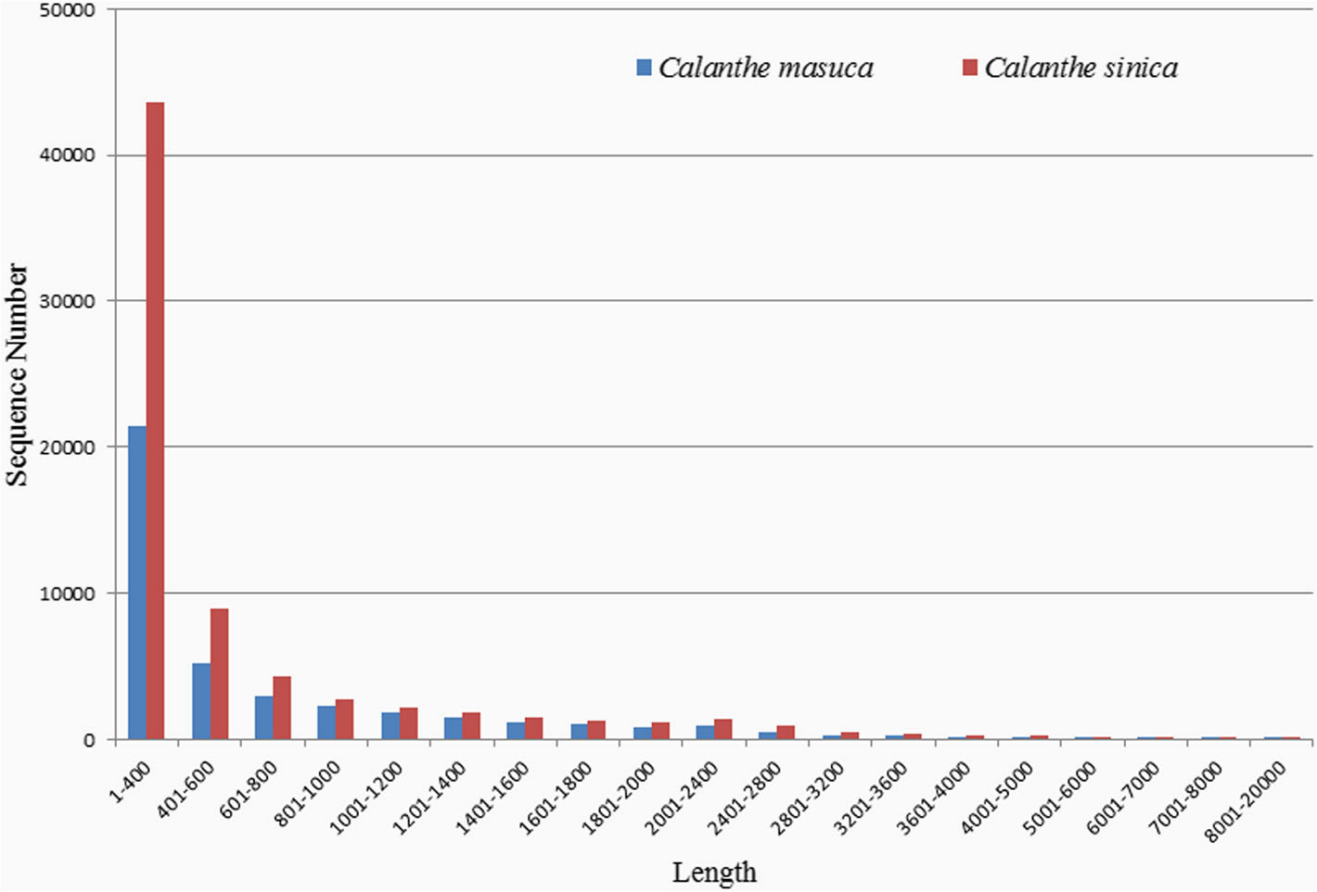
Sequence length distribution of the assembled unigenes of *C. masuca* and *C. sinica*. The x-axis indicates sequence length, and the y-axis indicates the number of unigenes

In total, 96,697,776/84,001,406 (*C. masuca*/*C. sinica*) reads with a length of 151 bp were obtained from the Illumina HiSeq sequencing platform, and 94,826,358/82,290,826 clean reads for the two species, respectively, were obtained after filtering out low-quality reads and adaptors. Using the de novo assembly tool Trinity, we obtained 40,916/71,618 unigenes with an average length of 703.67/625.38 bp, and the N50 values were 1,196/1,086 bp, respectively (Table 1). With the RBH method, 25,152 pairs of putative orthologous genes between the two species were defined (Additional file 2). The distributions of unigene size for both species are shown in Fig. 2. The GC contents for C. *masuca* and *C. sinica* unigenes were 44.44%/43.06%, respectively.

**Table 1:** Summary of the assembled transcripts of *C. sinica* and *C. masuca*

	*C. masuca*		*C. sinica*	Transcripts
	Unigenes	Transcripts	Unigenes	
Number of sequences	40,916	50,112	71,618	90,173
Total nucleotide bases	28,791,330	38,475,376	44,788,570	64,336,080
GC content (%)	44.44	44.27	43.06	43.00
Maximum length (bp)	12,973	12,973	16,684	16,684
Average length (bp)	703.67	767.97	625.3	713.47
N50	1,196	1,296	1,086	1,285

### Functional annotation and classification

All unigenes of both species were annotated against five databases (Pfam, KEGG, String, Swiss-Prot and NR databases) using BLASTx alignment with an E-value threshold of 1E-5 (Table 2). In total, 40,916/71,618 unigenes were obtained in *C. masuca* and *C. sinica*, of which 23,932 (58.5%)/28,037 (39.1%) unigenes of the two species, respectively, returned values when searched against the NR nucleotide database. E-values and similarity distributions of unigenes annotated in the NR database can be found in Table 3. The top three species against which BlastX hits could be identified for unigenes of the two *Calanthe* species were *Phoenix dactylifera* (13360, 32.65%/13151, 18.36%) (unigenes number, the percentage of the hit unigenes), *Vitis vinifera* (1422, 3.48%/2389, 3.34%) and *Oryza sativa* (590, 1.44%/796, 1.11%) (see Additional file 3 for matched species).

**Table 2:** The annotation list of *C. masuca* and *C. sinica*

	Pfam	KEGG	String	Swiss-Prot	NR
*C. masuca*	13,336	9,851	8,971	15,328	23,932
(%)	32.6	16.6	24.1	37.5	58.5
*C. sinica*	14,932	9,717	11,088	16,767	28,037
(%)	20.8	13.6	33.5	15.5	39.1

**Table 3:** E-value and similarity distribution of unigenes annotated in the NR database

E-value	*C. masuca* (n)	*C. sinica* (n)
0	12,736	13,980
0 to 1E-30	3,315	3,542
1E-30 to 1E-20	2,996	3,422
1E-20 to 1E-10	3,281	4,499
1E-10 to 1E-5	1,604	2,594
Similarity	(n)	(n)
20% to 40%	4	17
40% to 60%	1,492	2,152
60% to 80%	10,309	13,113
80% to 100%	12,127	12,755

### COG and GO classification

**Fig. 3:**
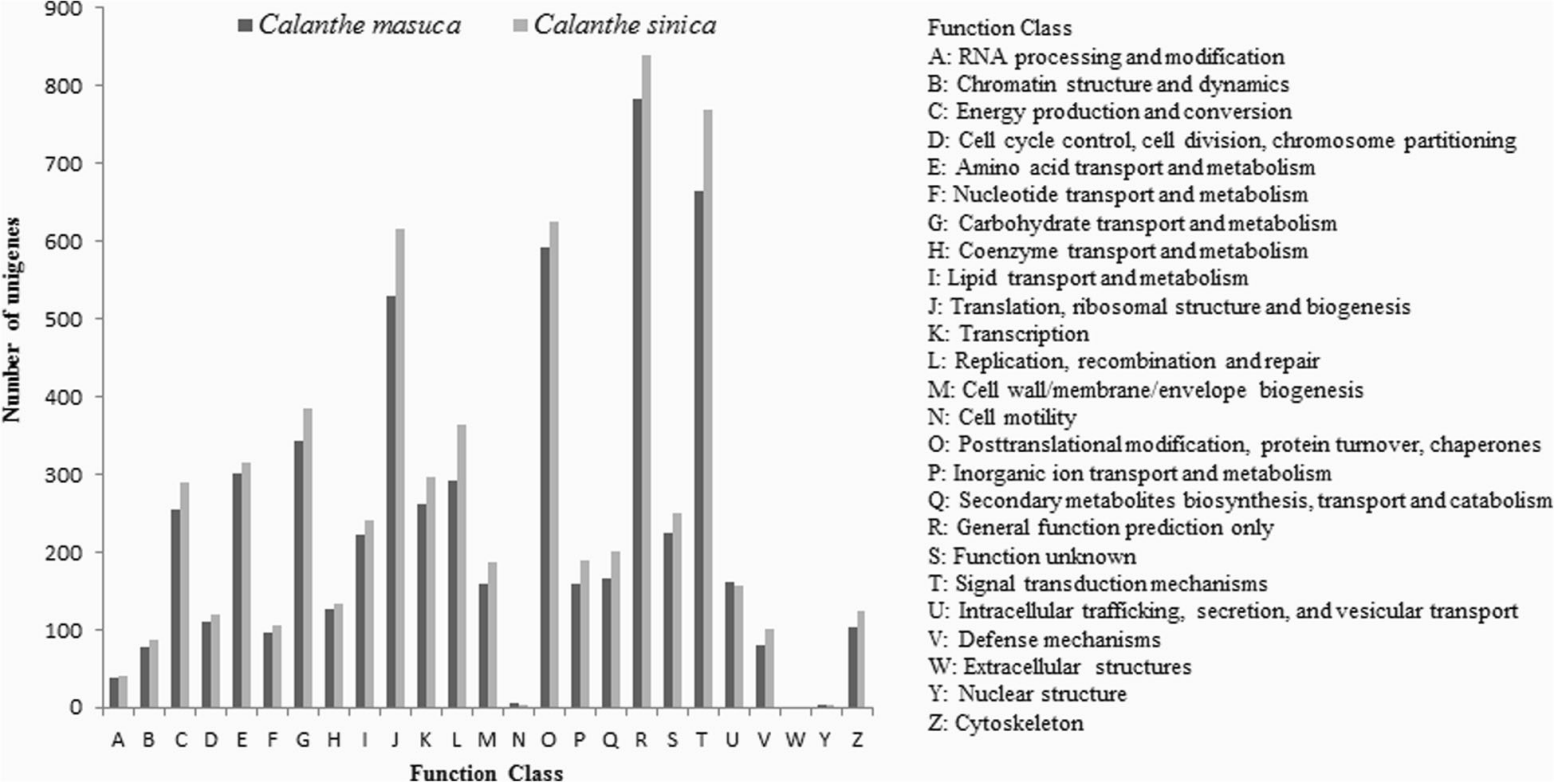
Clusters of orthologous group classification of *C. masuca* and *C. sinica*

By sequence comparison with genes from COG (Clusters of Orthologous Groups of proteins), we found that 9,851 unigenes (24.1% out of all unigenes) of *C. masuca* and 11,088 unigenes (15.5%) of *C. sinica*, respectively, could be classified into 26 categories (Fig. 3). The largest groups for both species were assigned to the cluster of ‘General function prediction’ (782, 1.91%/838, 1.17%), followed by ‘Signal transduction mechanisms’ (663, 1.62%/768, 1.07%), ‘Posttranslational modification, protein turnover, chaperones’ (593, 1.45%/624, 0.87%), ‘Translation, ribosomal structure and biogenesis’ (528, 1.29%/615, 0.86%), and ‘Carbohydrate transport and metabolism’ (344, 0.84%/385, 0.54%). The smallest sets were ‘Cell motility’ (6, 0.01%/4, 0%), ‘Nuclear structure’ (1, 0%/1, 0%) and ‘Extracellular structures’ (0, 0%/0, 0%).

**Fig. 4:**
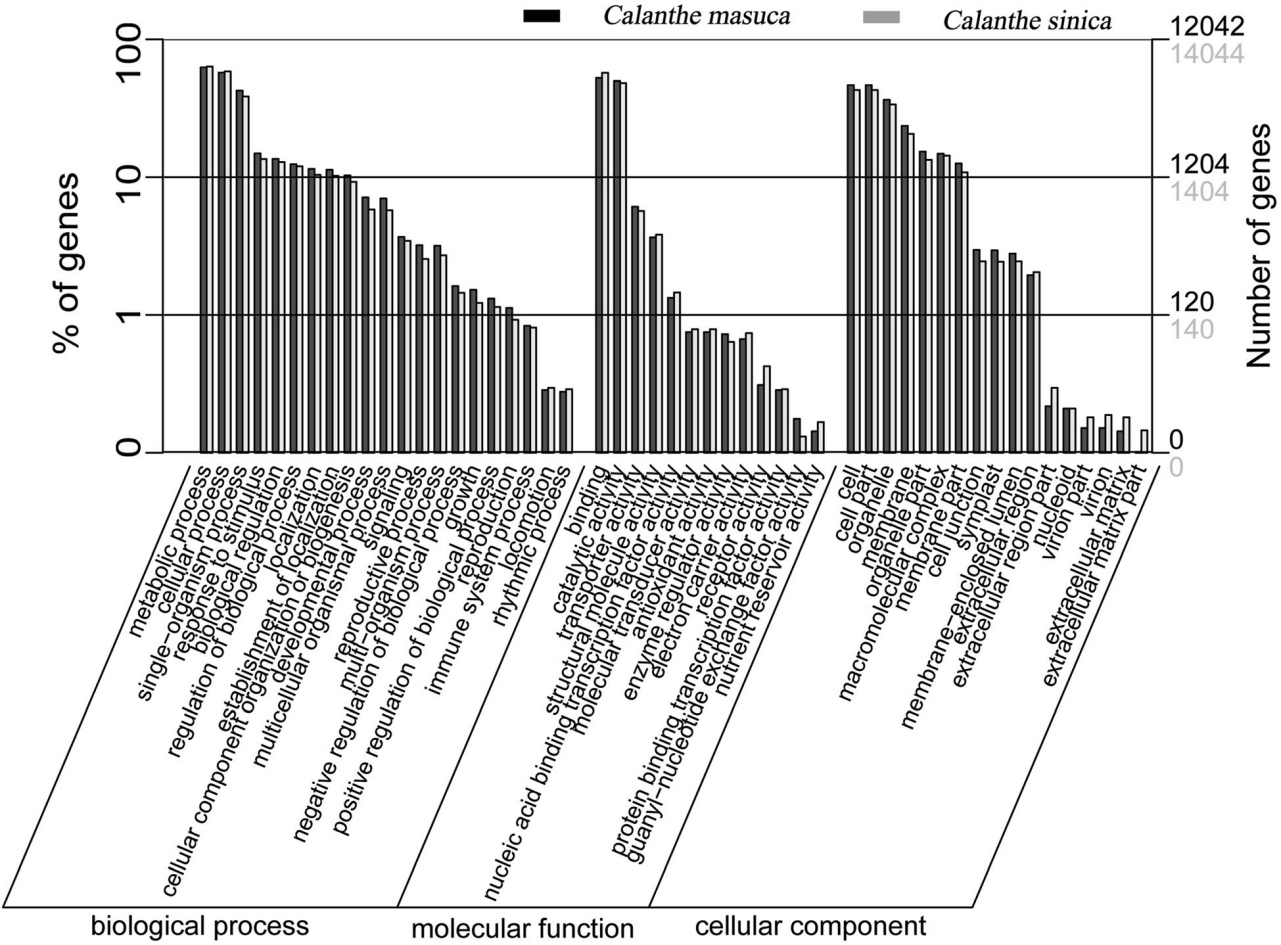
Gene ontology (GO) annotations of unigenes. The results are summarized into three main categories: biological processes, cellular components and molecular function

We then mapped our unigenes of the two species onto the annotation terms of the three categories in the GO classification system, i.e., biological processes, cellular components, and molecular function. A total of 12,042 (29.43%)/14,044 (19.61%) unigenes in *C. masuca* and *C. sinica*, respectively, were annotated to have at least one GO term (Fig. 4). The results showed a high degree of consistency in GO analysis between the two species. The top three groups with most gene members in the category of biological process were metabolic processes (7,509, 18.35%/8,879, 12.40%), cellular processes (6,890, 16.84%/8,198, 11.45%) and single-organism processes (5,121, 12.52%/5,407, 7.55%); the top three largest groups in the category of cellular components were cell (5,609, 13.71%/6,031, 8.42%), cell parts (5,609, 13.71%/6,031, 8.42%), and organelles (4,388, 10.72%/4,736 6.61%). We annotated most genes in both species with molecular functions of binding (6,312, 14.65%/8,016, 11.19%), catalytic activity (5,996, 14.65%/6,763, 9.44%), and transporter activity (726, 1.77%/788, 1.10%).

### A preliminary investigation into the evolutionary pattern of genes in *Calanthe masuca* and *C. sinica*

With the transcriptome data of two closely related *Calanthe* species available, it is possible for us to investigate the conservation and divergence pattern of genes in this genus, especially in the lineage represented by *C. masuca* and *C. sinica*. To more accurately define orthologous gene pairs, we employed the software Inparanoid (version 4.1; [26]) to define orthologous gene groups between the two species studied here, using *P. equestris* as the outgroup species. We then calculated the Ks, Ka, and Ka/Ks for each possible orthologous gene pair between *C. masuca* and *C. sinica*. We obtained the Ka, Ks, and Ka/Ks values for 10,329 orthologous gene groups between the two species, comprising 12,508 *C. masuca* unigenes and 11,493 *C. sinica* unigenes. The Ka/Ks value measures the average selective pressure of the two compared protein-coding sequences since their divergence. To gain preliminary insights into the distinct evolutionary pattern of genes involved in different functional pathways in *Calanthe*, we divided the genes in each species into 5 different groups according to their Ka/Ks values, namely, the group with strong purifying selection (Ka/Ks ≤ 0.5, abbreviated as SP), the group with intermediately purifying selection (0.5 < Ka/Ks ≤ 0.9, IP), the group with relaxed or nearly relaxed selection (0.9 < Ka/Ks ≤ 1.1, R), the group with intermediately positive or Darwinian selection (1.1 < Ka/Ks ≤ 1.5, ID), and the group with strong Darwinian selection (Ka/Ks > 1.5, SD).

The top enriched GO terms in each corresponding group were quite consistent between the two species (Additional file 4). For example, in the SP group of genes in both species, the biological process ‘oxidation-reduction process’ (GO: 0055114) stood out as the most enriched GO term, with 348 and 332 associated unigenes in *C. masuca* and *C. sinica*, respectively. Similarly, ‘folic acid-containing compound biosynthetic process’ and ‘phosphate ion transport’ were among the top 3 enriched GO terms in the IP group in both species. A systematic comparison demonstrated that, in most cases, the shared enriched GO terms between corresponding gene groups of the two species outnumbered GO terms specifically enriched in each species (Table 4), which further validated the assembly and annotation of our RNA-seq data. Notably, the biological process ‘aldehyde biosynthetic process’ (GO: 0046184) was among the top three enriched GO terms in both species, implying fast-evolving indirect plant defence-responsive factors that could help *Calanthe* species adapt to local environments [36].

**Table 4:** Comparison of enriched GO terms among genes of different classes in *C. masuca* and *C. sinica*

GO category	Gene group	*C. masuca*-specific	*C. sinica*-specific	shared
BP	SP	20	20	30
	IP	8	13	7
	R	12	11	20
	ID	9	7	9
	SD	7	9	11
MF	SP	12	18	32
	IP	3	6	13
	R	1	4	5
	ID	5	4	3
	SD	3	4	5
CC	SP	9	10	13
	IP	1	7	3
	R	1	2	2
	ID	3	1	2
	SD	3	0	2

*Abbreviations*: *BP* biological process, *MF* molecular function, *CC* cellular component. gene classes, *SP* strong purifying selection (Ks ≤ 0.5), *IP* intermediate purifying selection (0.5 < Ks ≤ 0.9), *R* relaxed or nearly relaxed selection (0.9 < Ks ≤ 1.1), *ID* intermediate Darwinian selection (1.1 < Ks ≤ 1.5), *SD*, strong Darwinian selection (Ks > 1.5). GO enrichment results were obtained using the R package topGO

**Fig. 5:**
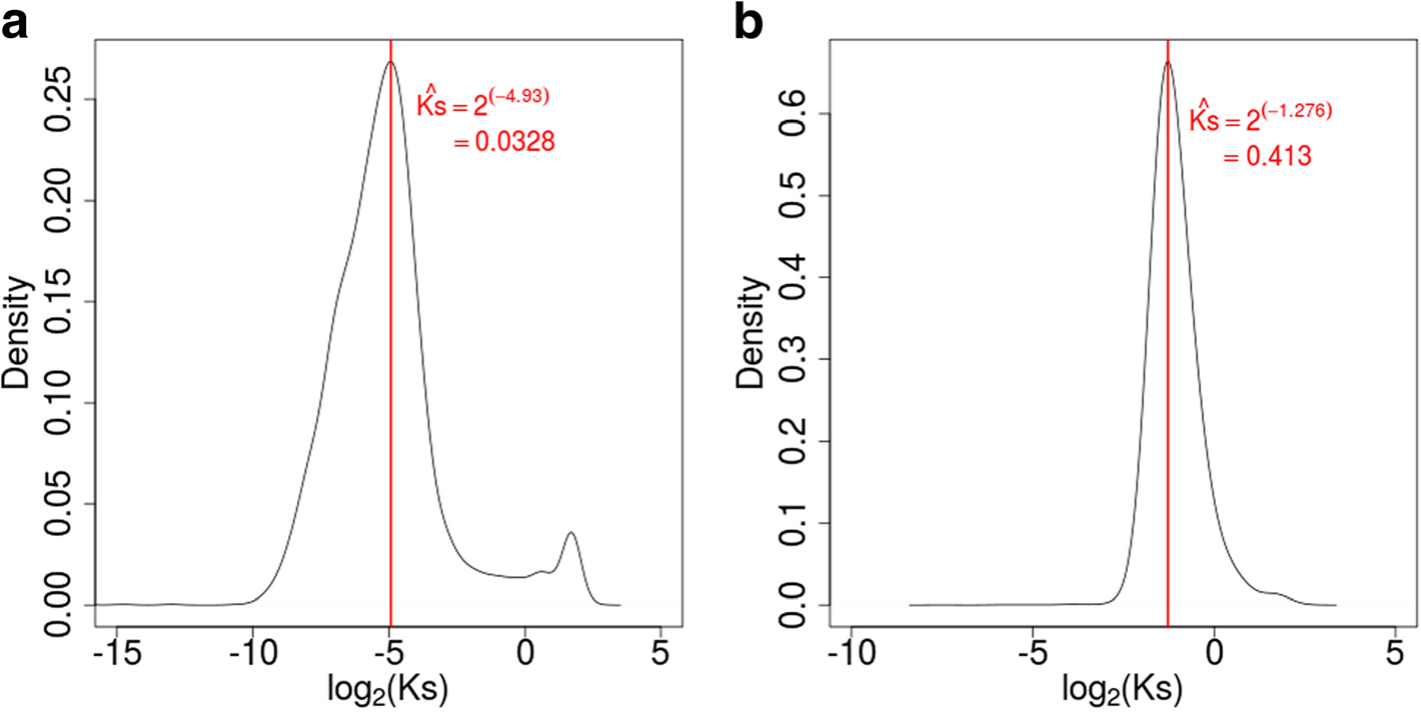
Determination of divergence time between *C. masuca* and *C. sinica*. The distribution of minimal synonymous substitutions per synonymous substation sites (Ks) of the orthologous gene groups between *C. masuca* and *C. sinica* (**a**) and between *Phalaenopsis equestris* and *Dendrobium catenatum* (**b**). The divergence time between *P. equestris* and *D. catenatum* determined in the publication [37] was used as real-time scale proportional to the measure of Ks

To understand the above evolutionary pattern in a real time-scale of species evolution, we set to estimate the divergence time between the two closely related *Calanthe* species. For a more robust estimation, we determined the minimum Ks values for each of 10,329 orthologous gene groups and determined the mode of the distribution of these Ks values at ~ 0.0328 (Fig. 5). Using the same method, we obtained orthologous gene groups between two epidendroid orchid species, i.e., *P. equestris* and *D. catenatum*, and calculated the Ks values for each orthologue pair. The mode of distribution of the minimum Ks values of the orthologous gene groups between *P. equestris* and *D. catenatum* was determined at 0.413 (Fig. 5). With the divergence time between these two epidendroid orchids estimated at ~ 43 Mya [37] and assuming that the non-synonymous substitution rates between the two epidendroid and the two *Calanthe* species were close, we proposed that *C. masuca* and *C. sinica* diverged from each other approximately 3.42 ± 1.86 Mya. This preliminary estimation of the divergence time between *C. masuca* and *C. sinica* could be improved in the future with more data and information available.

### SSR identification, screening of variations and validation

**Fig. 6:**
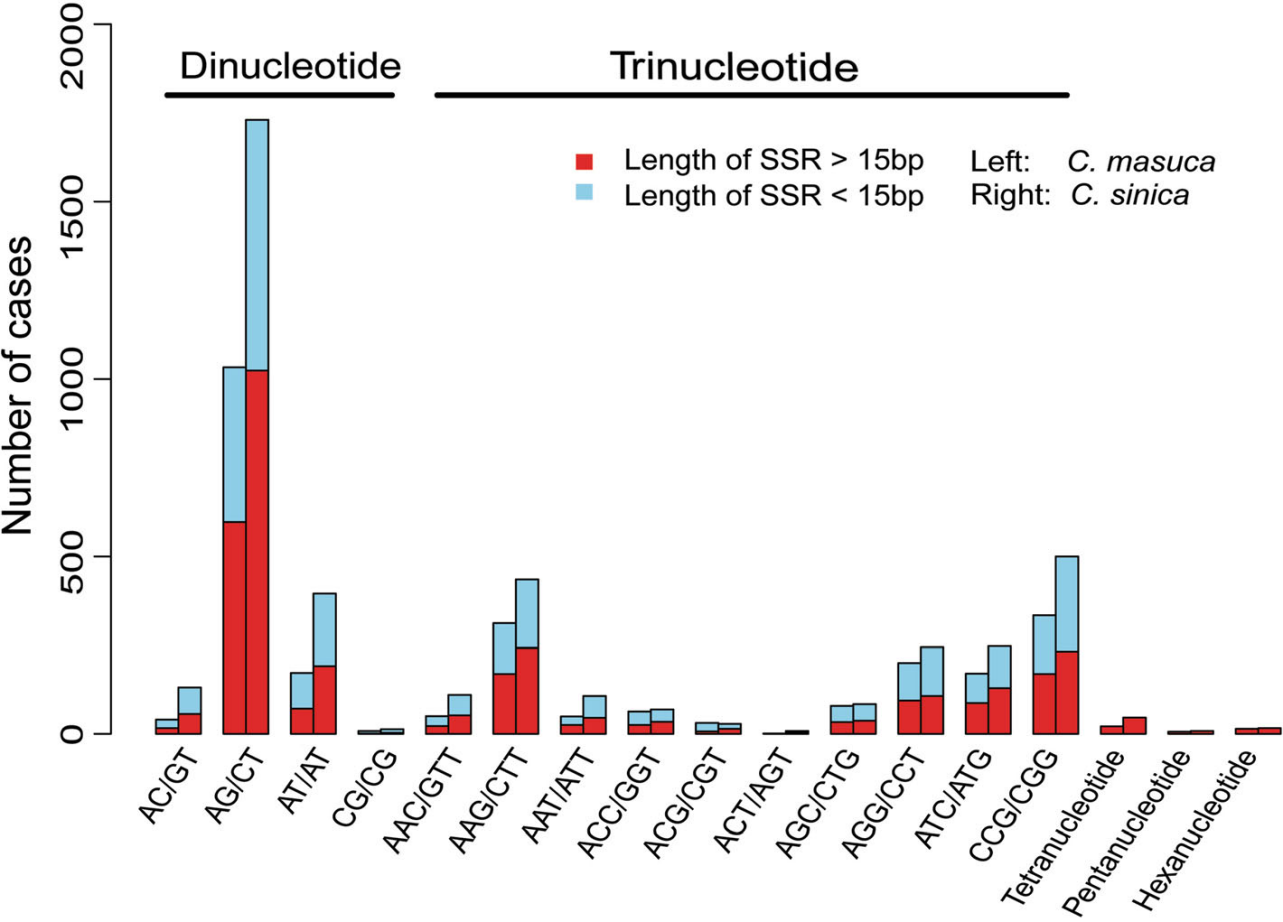
The distribution of SSR type in the *C. masuca* and *C. sinica* transcriptomes. The SSR repeat type of mononucleotides was excluded

In total, 3,764 putative SSRs in *C. masuca* and 7,189 SSRs in *C. sinica* were identified. The majority of the SSRs in both species were either dinucleotides (1,137 in *C. sinica* and 2,058 in *C. masuca*) or trinucleotides (1,118 in *C. sinica* and 1,714 in *C. masuca*), with longer motifs accounting for only a small proportion (Fig. 6). Finally, 2,019/3,865 primer pairs were successfully designated by Msatcommander for the two species (Additional file 5). In these primers, we screened 197 polymorphic SSR loci according to 25,152 pairs of putative orthologous genes identified by the method of reciprocal best hit (Additional file 5). In addition, 129 SSR loci remained after mononucleotides were excluded (Additional file 5).

### Validation of SSR markers

To test the amplification quality and the polymorphisms for the preliminary analysis of the 129 SSR markers, a single sample, JX1 (*C. masuca*), was used, and it was found that 75 SSRs resulted in clear peaks with little disturbance.

**Fig. 7:**
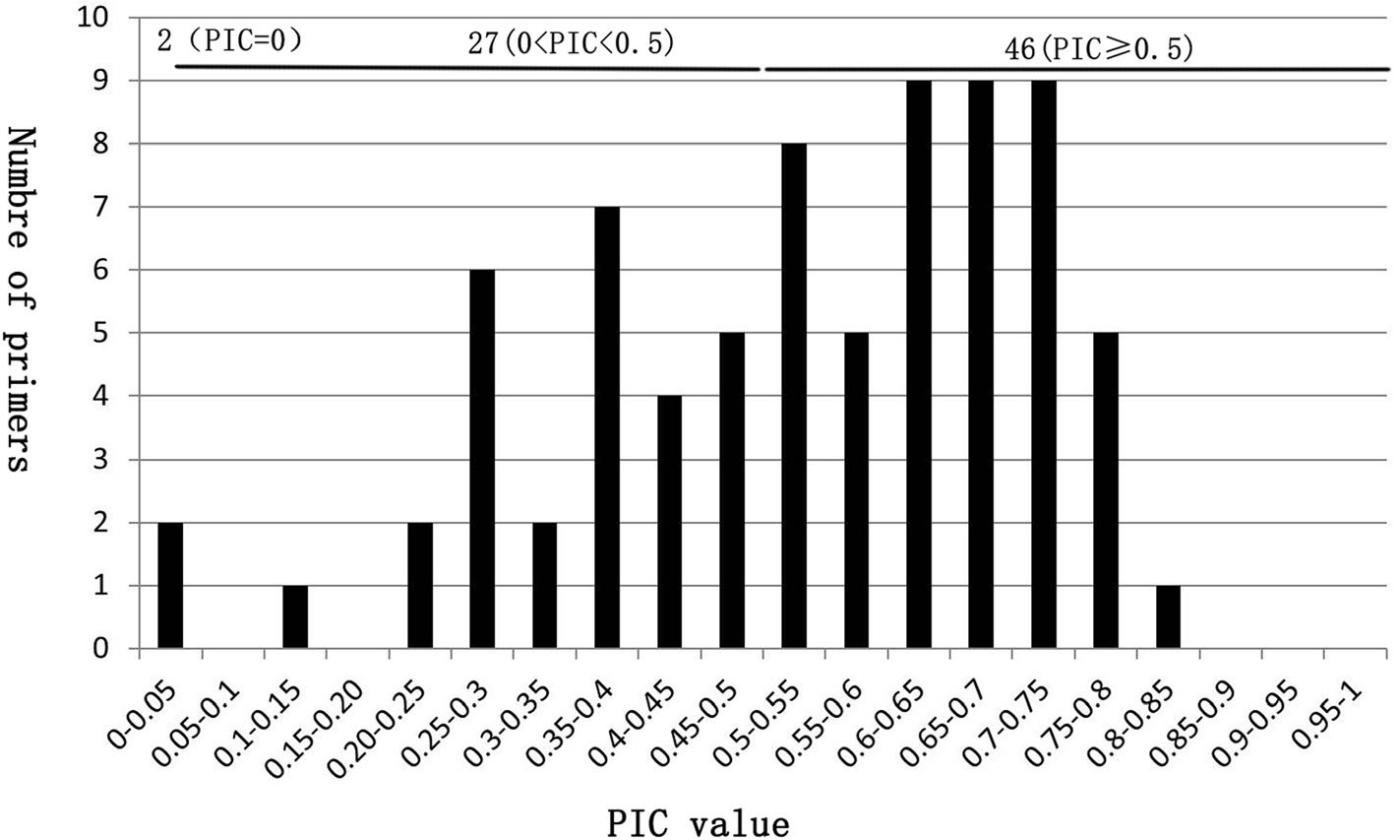
The distribution of polymorphism information content (PIC)

We then performed polymorphic analysis using eight individuals from each population collected from Yunnan, Jiangxi, Napo, and Guangxi in China (Additional file 1). We found that 73 of the 75 examined loci were polymorphic (97.6%) (Fig. 7 and Additional file 6). The two monomorphic loci regarding these 8 individuals could represent polymorphic sites of the subpopulations from which we collected the samples for RNA sequencing. Although potentially polymorphic for other uninspected populations, the two primers were excluded from further analysis for convenience. Nonetheless, 44 out of the 73 loci were found to have a relatively high polymorphic level (PIC≥0.5) and could thus be used in population genetic studies.

To validate the reliability of the primers of the identified polymorphic loci, 13 primers with high PIC values (PIC≥0.5) were randomly selected to amplify a large number of populations and individuals (other 72 individuals). We found that all primers generated high PIC (PIC≥0.5) values (Additional file 7), confirming that the primers could be used in future genetic analysis of *Calanthe*.

### Universality evaluation of SSR primers

The polymorphic SSR markers screening presented above was performed by comparing the transcriptomes of individuals from subpopulations in the two *Calanthe* species; henceforth, a limited number of polymorphic SSR loci were identified (Additional file 8). To develop a larger number of candidate polymorphic SSR loci, we performed in silico PCR analysis. For the two *Calanthe* species, the identified primer pairs were compared between them, and both were compared against two orchids with published genomic sequences, *P. equestris* [27] and *D. catenatum* [34]*.* For stringency, all hits with mismatches against the two non-*Calanthe* orchids were excluded. As a result, among all the 2,019 *C. masuca* primer pairs, we identified 1,156 (57.3%) pairs that could be used for *C. sinica*, and in contrast, only 13 (0.6%) primers for *D. catenatum* were identified. Similarly, among the 3,865 primers of *C. sinica*, 869 (22.5%) could be used for *C. masuca*, and only 14 (0.4%) could be used for *D. catenatum*. We failed to identify any primers from the two *Calanthe* species for *P. equestris*. The matching rate of in silico PCR effectively reflects the genetic relationship of these species, as well as the number of transcripts recovered by our RNA-seq analysis.

## Discussion

*Calanthe masuca* and *C. sinica* are orchid species that play important roles in horticulture. For these two species, with genome size of about 11.23 pg whole genome sequencing is difficult in short timescales. Transcriptome sequencing is hence an important tool for molecular marker identification and gene discovery [38]. In this study,we sequenced the transcriptome of *C. masuca* and *C. sinica*, to identify useful SSR markers and obtain transcriptomic information for further studies. Plant genomic analyses can be performed using NGS technologies to determine whether plants have a complete genome sequence. NGS has been used for de novo transcriptome sequencing and assembly in many organisms, including orchids [39–42]. In this study, 11 Gbp of data were obtained from leaf tissue of each species and assembled into unigenes.

To a certain extent, the length of the assembly sequence reflected the quality of the transcriptome obtained from the Illumina platform [43]. The average unigene length in this study was shorter than in *Phalaenopsis aphrodite* (875 bp) but longer than in *Cymbidium sinensis* (612 bp) [41, 42]. This means that the transcriptome data for *Calanthe* in this study was assembled efficiently.

### The conservation and divergence of *Calanthe masuca* and *C. sinica* genes

A previous phylogenetic study suggested that *C. masuca* and *C. sinica* were closely related to each other [1, 2]. They are winthin *sect. Calanthe* series *sylvatica*. *C. masuca* and *C. sinica* are the only two species in this series existing in China. They have many common phenotypic characteristics such as long spur, warty calli on the labellum and similar shape of lip midlobe, but still exhibit many phenotypic differences, such as shape of spur. The GO enrichment analysis carried out for each class of genes separately gave similar results in most cases, i.e., We observed much overlap in the enriched GO terms between the corresponding gene classes of the two species. However, we also noted interesting clues in species-specifically enriched GO terms. The number of specifically enriched GO terms of each category in the ID group of genes in *C. sinica* was also significantly greater than that in *C. masuca*, further supporting the possibility of specific local adaptation for *C. sinica* after its divergence with *C. masuca*. These distinct evolutionary patterns could have occurred on a relatively short time scale, within ~ 3.42 million years. These observations could be validated and/or broadened in future bio-geographical and functional studies.

### EST-SSR prediction and validation

SSRs are tandem repeats of short nucleotide motifs with polymorphisms of a certain length that are spread throughout the genome [44]. EST-SSRs have many intrinsic advantages over genomic SSR markers due to their higher transferability among related species [45]. EST-SSR frequency is dependent on several factors, such as genome structure or composition, arithmetical method for SSR detection, and the parameters for exploration of microsatellites [38]. In the two transcriptomes generated in this study, 3,764/7,189 putative SSRs were predicted that differed in SSR number. This may be the cause of differences in genome structure or composition. In both transcriptomes, AG/CT had the highest frequency, as shown in other species [38, 39, 46], and CG/CG had the lowest frequency. In this study, we performed RBH to screen out 25,152 pairs of putative orthologous genes. After searching for SSR loci among these genes, SSR loci with differences between the two transcriptomes were manually identified. This method resulted in SSR loci differing in at least two individuals, which ensured the development of SSR markers with a high level of success. However, the selected alleles may not truly be polymorphic due to the possibility of errors in orthologue prediction. This is why there were two monomorphic loci in this experiment. Even so, this processing can greatly improve the efficiency of screening polymorphic SSR loci.

In this study, we identified 44 loci with high PIC values, and 13 polymorphic SSR loci were randomly chosen for testing from 96 *Calanthe* individuals, demonstrating their reliability. The remaining 34 loci may also be reliable. These loci can be used in further studies, such as for investigating genetic linkage mapping, germplasm characterization, fingerprinting, and genetic diversity.

### Universality evaluation of SSR primers

Using in silico PCR against genome sequences is a practical method for preselecting primers from transcriptomes because the existence of a large intron between primer pairs will greatly affect the performance of PCR amplification [39]. However, this analysis resulted in no identical SSR loci between *P. equestris* and *Calanthe*, possibly because of their distant relationship. *D. catenatum* had 13/14 hits with *C. masuca*/*C. sinica*. If genome data from other species closely related to *Calanthe* were sequenced and included in the analysis, more hits would be identified.

## Conclusions

The RNA-seq and de novo transcriptome assembly of two closely related *Calanthe* species, *C. masuca* and *C. sinica*, was conducted. 13 randomly selected primers with a high polymorphism level generated a high level of PIC. A divergence time of 3.42 ± 1.86 Mya was determined between the two species according to Ks. In the future, genetic diversity studies can be conducted in the two *Calanthe* species and even other closely related species in this genus. Hence some protection strategy can be developed to save these species.

## Additional files

Additional file 1: **Table S1.** Materials used in this article. (DOCX 14 kb)

Additional file 2: **Table S2.** List of Reciprocal Best Hits of *C. masuca* and *C. sinica. (XLSX 741 kb)*

Additional file 3: **Table S3.** Proportion of matched unigenes in the NR database. (DOCX 15 kb)

Additional file 4: **Table S4.** GO annotations of unigenes with Ka/Ks and the results of following enrichment analysis. (XLSX 100 kb)

Additional file 5: **Table S5.** List of SSR primer pairs derived from two species and 129 selected SSR primers. (XLSX 1354 kb)

Additional file 6: **Table S6.** The statistical parameters of 75 validated primer pairs using eight individuals of each species. (XLSX 18 kb)

Additional file 7: **Table S7.** The statistical parameters of 13 validated primer pairs using 96 individuals. (XLSX 12 kb)

Additional file 8: **Table S8.** In silico PCR analysis. Using the primers of *C. masuca* and *C. sinica* against the unigene of each other and against *Phalaenopsis equestris* and *Dendrobium catenatum*. (XLSX 114 kb)

## Abbreviations

e-PCR: Electronic PCR
EST-SSR: Expressed sequence tag-SSR
*He*: Expected heterozygosity
*Ho*: Observed heterozygosity
*Na*: Number of alleles
PIC: Polymorphism information content
RBH: Reciprocal best hits algorithm
SSR: Polymorphic microsatellite marker
