# Supplementary material for: Development of polymorphic microsatellite markers by using de novo transcriptome assembly of Calanthe masuca and C. sinica (Orchidaceae)
Source: BMC Genomics. 2018 Nov 6;19:800. doi: 10.1186/s12864-018-5161-4 (PMC6219035; doi:10.1186/s12864-018-5161-4)
Supplement: Supplementary file 1 — Table S1. Materials used in this article. (DOCX 14 kb) [file 12864_2018_5161_MOESM1_ESM.docx]

**Additional file 1 Table S1. Plant Materials Used in this article**

| Species | Populations | Location | GPS | Individuals |
| --- | --- | --- | --- | --- |
| Transcriptome analysis |  |  |  |  |
| *Calanthe masuca* |  | Shanghai Chenshan Botanical Garden |  | 1 |
| *Calanthe sinica* |  | Shanghai Chenshan Botanical Garden |  | 1 |
| Polymorphism analysis |  |  |  |  |
| *Calanthe masuca* | JX | Jiulianshan, Jiangxi Province, China | N 24°37'24.63" E 114°33'53.46" | 32 |
| *Calanthe masuca* | GXJX | Jinxiu, Guangxi Province, China | N 24°13'11.51" E 110°15'50.91" | 20 |
| *Calanthe masuca* | TW | Alishan, Jiayi County , Taiwan | N 23°24'57.98" E 120°46'19.83" | 15 |
| *Calanthe sinica* | GXNP | Napo County, Guangxi Province, China | N 23°29'58.92" E 105°46'34.73" | 10 |
| *Calanthe triplicata* | YN | Xishuangbanna,Yunnan, China | N 21°36'54.55" E 101°34'47.04" | 19 |
