# Supplementary material for: Development of polymorphic microsatellite markers by using de novo transcriptome assembly of Calanthe masuca and C. sinica (Orchidaceae)
Source: BMC Genomics. 2018 Nov 6;19:800. doi: 10.1186/s12864-018-5161-4 (PMC6219035; doi:10.1186/s12864-018-5161-4)
Supplement: Supplementary file 3 — Table S3. Proportion of matched unigenes in the NR database. (DOCX 15 kb) [file 12864_2018_5161_MOESM3_ESM.docx]

**Additional file 3: Table S3.** **Proportion of matched unigenes in the NR database**

| Species | Number of matched unigenes (*C. masuca*) | Percentage of matched unigenes (*C. masuca*) | Species | Number of matched unigenes (*C. sinica*) | Percentage of matched unigenes (*C. sinica*) |
| --- | --- | --- | --- | --- | --- |
| *Phoenix dactylifera* | 13360 | 32.65% | *Phoenix dactylifera* | 13151 | 18.36% |
| *Vitis vinifera* | 1422 | 3.48% | *Vitis vinifera* | 2389 | 3.34% |
| *Oryza sativa* | 590 | 1.44% | *Oryza sativa* | 796 | 1.11% |
| *Theobroma cacao* | 485 | 1.19% | *Theobroma cacao* | 655 | 0.91% |
| *Setaria italica* | 317 | 0.77% | *Malus domestica* | 462 | 0.65% |
| *Morus notabilis* | 310 | 0.76% | *Morus notabilis* | 437 | 0.61% |
| *Populus trichocarpa* | 279 | 0.68% | *Prunus persica* | 413 | 0.58% |
| *Prunus persica* | 274 | 0.67% | *Glycine max* | 408 | 0.57% |
| *Malus domestica* | 265 | 0.65% | *Setaria italica* | 346 | 0.48% |
| *Glycine max* | 259 | 0.63% | *Populus trichocarpa* | 322 | 0.45% |
| *Zea mays* | 248 | 0.61% | *Prunus mume* | 287 | 0.40% |
| *Sorghum bicolor* | 239 | 0.58% | *Zea mays* | 272 | 0.38% |
| *Jatropha curcas* | 221 | 0.54% | *Sorghum bicolor* | 268 | 0.37% |
| *Prunus mume* | 203 | 0.50% | *Arabidopsis thaliana* | 249 | 0.35% |
| *Ricinus communis* | 197 | 0.48% | *Ricinus communis* | 222 | 0.31% |
| *Citrus clementina* | 189 | 0.46% | *Medicago truncatula* | 217 | 0.30% |
| *Eucalyptus grandis* | 185 | 0.45% | *Jatropha curcas* | 208 | 0.29% |
| *Oryza brachyantha* | 181 | 0.44% | *Citrus sinensis* | 206 | 0.29% |
| *Amborella trichopoda* | 166 | 0.41% | *Oryza brachyantha* | 197 | 0.28% |
| *Coffea canephora* | 149 | 0.36% | *Citrus clementina* | 189 | 0.26% |
| other | 3775 | 9.23% | other | 5447 | 7.61% |
| species unknown | 618 | 1.51% | species unknown | 896 | 1.25% |
